# Programmed Death Ligand-1 (PD-L1) Is an Independent Negative Prognosticator in Western-World Gallbladder Cancer

**DOI:** 10.3390/cancers13071682

**Published:** 2021-04-02

**Authors:** Thomas Albrecht, Fritz Brinkmann, Michael Albrecht, Anke S. Lonsdorf, Arianeb Mehrabi, Katrin Hoffmann, Yakup Kulu, Alphonse Charbel, Monika N. Vogel, Christian Rupp, Bruno Köhler, Christoph Springfeld, Peter Schirmacher, Stephanie Roessler, Benjamin Goeppert

**Affiliations:** 1 Institute of Pathology, Heidelberg University Hospital, 69120 Heidelberg, Germany; Thomas.Albrecht@med.uni-heidelberg.de (T.A.); Fritz.Brinkmann@med.uni-heidelberg.de (F.B.); Alphonse.Charbel@med.uni-heidelberg.de (A.C.); Peter.Schirmacher@med.uni-heidelberg.de (P.S.); Stephanie.Roessler@med.uni-heidelberg.de (S.R.); 2 Liver Cancer Center Heidelberg (LCCH), 69120 Heidelberg, Germany; Arianeb.Mehrabi@med.uni-heidelberg.de (A.M.); Katrin.Hoffmann@med.uni-heidelberg.de (K.H.); Christian.Rupp@med.uni-heidelberg.de (C.R.); Bruno.Koehler@med.uni-heidelberg.de (B.K.); Christoph.Springfeld@med.uni-heidelberg.de (C.S.); 3 European Center for Angioscience (ECAS), Medical Faculty of Mannheim, Heidelberg University, 68167 Mannheim, Germany; Michael.Albrecht@stud.uni-heidelberg.de; 4 Department of Dermatology, Heidelberg University Hospital, 69120 Heidelberg, Germany; anke.lonsdorf@med.uni-heidelberg.de; 5 Department of General, Visceral and Transplantation Surgery, Heidelberg University Hospital, 69120 Heidelberg, Germany; Yakup.Kulu@med.uni-heidelberg.de; 6 Diagnostic and Interventional Radiology, Thoraxklinik at Heidelberg University Hospital, 69126 Heidelberg, Germany; Monika.Vogel@med.uni-heidelberg.de; 7 Department of Internal Medicine IV, Gastroenterology and Hepatology, Heidelberg University Hospital, 69120 Heidelberg, Germany; 8 National Center for Tumor Diseases, Department of Medical Oncology, Heidelberg University Hospital, 69120 Heidelberg, Germany

**Keywords:** programmed cell death ligand-1, PD-L1, gallbladder cancer, biomarkers, tumor, gastrointestinal neoplasms, immune evasion, liver neoplasms

## Abstract

**Simple Summary:**

Gallbladder cancer (GBC) is an aggressive malignancy with poor prognosis. Currently, therapeutic options are mostly limited to palliative chemotherapy. Considering the advances of immunotherapy, we assessed the expression of the programmed cell death ligand-1 (PD-L1) as the most widely used predictive marker for immunotherapy response in a large Western-world GBC cohort. Additionally, we quantified the expression of the T-cell immunoreceptor with Ig and ITIM domains TIGIT/CD155 axis as an emerging immune checkpoint. Our results indicate that PD-L1 is heterogeneously expressed in Western-world GBC and associated with distinct histomorphological tumor subtypes and increased immune cell densities. We show that a high tumoral PD-L1 expression is a significant negative prognosticator. In a subset of patients, we identified expression of TIGIT in scattered immune cells, which correlated with tumoral expression of its ligand CD155. Our results suggest a subset of GBC patients to be candidates for immunotherapy via (combined) PD-L1 and TIGIT/CD155 inhibition.

**Abstract:**

Inhibition of the programmed cell death protein-1/ligand-1 (PD-1/PD-L1) axis has opened a new era in the treatment of solid cancers. However, there is no data on the expression and relevance of PD-L1 in Western gallbladder cancer (GBC). We assessed PD-L1 immunohistochemically in 131 GBC patients as Tumor Proportion Score (TPS), Immune Cell Score (IC) and Combined Positivity Score (CPS). Tumor cells expressed PD-L1 in a subset of 14.7% GBC patients at a TPS cut-off of 1%. Higher PD-L1 levels above 10% and 25% TPS were reached in 4.7% and 3.1% of GBC cases, respectively. At a 10% cut-off, TPS was associated with distinct histomorphological subtypes and correlated with poor tumor differentiation. Survival analysis revealed a TPS above 10% to be a highly significant and independent negative prognosticator in GBC. PD-L1 expression was associated with increased CD4^+^, CD8^+^ and PD-1^+^ immune cell densities. In 14.8% of the cases, scattered immune cells expressed T-cell immunoreceptor with Ig and ITIM domains (TIGIT), which was correlated to tumoral expression of its ligand CD155. We here show that a high PD-L1 expression confers a negative prognostic value in Western-world GBC and highlight the TIGIT/CD155 immune checkpoint as a potential new target for GBC immunotherapy.

## 1. Introduction

Gallbladder cancer (GBC) is the most common malignancy of the biliary tract, characterized by an extremely aggressive biological behavior [1]. The majority of patients present with advanced disease already at time of diagnosis, rendering them ineligible for surgical resection as the only potentially curative treatment option. Since there are no established targeted therapies for GBC, gemcitabine/cisplatin-based chemotherapy still remains the backbone of systemic treatment for those patients, despite very limited beneficial effect [2]. As such, in contrast to a variety of other solid cancer types, GBC still confers a largely unchanged, abysmal prognosis with a median survival of less than one year [3].

Recent studies revealed that many tumors specifically modulate physiologic immune homeostasis in order to escape antitumor immune responses [4,5,6]. Dysregulation of central immune-inhibitory and -stimulating molecules, known as immune checkpoints, seems to significantly contribute to immune evasion in numerous malignancies. Among those molecules, disruption of the programmed cell death protein-1 (PD-1)/programmed death ligand-1 (PD-L1) axis was demonstrated to be of particular relevance for tumor progression and survival [7,8]. Physiologically, binding of PD-L1 to its receptor PD-1 on activated immune cells negatively regulates T cell-driven immune responses in peripheral tissues, mediating immune tolerance. There is a growing body of evidence that PD-L1 is constitutively expressed by a variety of solid and hematologic malignancies, which thus escape immunosurveillance [9]. Therapeutic blockage of the PD-1/PD-L1 checkpoint has proven significant clinical benefit in many advanced solid malignancies like melanoma and non-small cell lung cancers and opened a new era of precision cancer medicine [10,11,12]. However, there is a significant subset of patients who do not benefit from this treatment, which in addition may be associated with severe adverse events [13]. If unselected, response rates to PD-1/PD-L1 inhibition in melanoma and non-small cell lung cancer are reportedly lower [14]. Analogous to other tailored therapies like inhibition of human epidermal growth factor receptor 2 (HER2), evaluation of predictive biomarkers on the individual’s tumor tissue prior to treatment initiation is therefore of pivotal importance. Immunohistochemical assessment of PD-L1 expression on tumor tissue is the first clinically validated predictive biomarker for immunotherapy that has translated into clinical practice [15,16]. Several scoring algorithms have been implemented with indication- and drug-specific cut-offs making predictive PD-1/PD-L1 testing an increasingly complex challenge in routine pathology [17].

Current knowledge on PD-L1 expression in GBC is scarce and based on three studies from regions with comparably high GBC incidences. Here, the frequency of tumor cell positivity for PD-L1 ranged from 18% in two studies on Asian cohorts to 23% determined in the study by Neyaz et al. conducted in India [18,19,20]. Since the regional differences in GBC incidence strongly suggest distinct genetic and environmental factors to drive tumorigenesis, these findings cannot be directly extrapolated to GBC of Western-World populations [21]. This is underpinned by our own clinical experience of a somewhat lower frequency of PD-L1 expression in GBC than previously reported.

The exceptionally malignant behavior and absence of effective therapies render GBC a potential candidate for immunotherapy. However, as of today not a single study on the expression of PD-1/PD-L1 in GBC has been performed in a Western-world country. In this study, we assessed PD-L1 expression in a large and comprehensive European GBC cohort encompassing 131 patients, including 74 cases with concomitant high-grade biliary intraepithelial neoplasia (BilIN). By application of stringent evaluation criteria, we thus aimed to provide a solid database of PD-L1 expression in Western GBC and to disclose potential associations with clinically relevant parameters. In addition, we investigated whether the recently identified T-cell immunoreceptor with Ig and ITIM domains (TIGIT)/CD155 immune checkpoint may contribute to immune exhaustion and represent a complement target for GBC immunotherapy.

## 2. Materials and Methods

### 2.1. Clinicopathological Cohort Characteristics

Surgical resection specimen of a number of 131 patients with a pathological diagnosis of primary carcinoma of the gallbladder were enrolled in this study. All patients underwent surgery at Heidelberg University Hospital between 1995 and 2016. Only radical cholecystectomy and right hemihepatectomy specimens were included; biopsies were omitted. The median age of the patients was 72.2 years. Approximately two thirds of the study cohort were female (67.2%). Patients with competing malignancies at the time of diagnosis were excluded. Only adenocarcinomas or adenosquamous carcinomas were enrolled in this study, including different histomorphological subtypes of adenocarcinoma (NOS, mucinous, signet ring, papillary, solid and intestinal). None of the patients received radiotherapy or chemotherapy prior to surgery. Tumors were re-staged according to the 8th TNM Classification of Malignant Tumours and classified following the World Health Organization (WHO) tumour classification system (*Digestive System Tumours*, 5th edition). Survival data were available for 104 patients. The use of tissue specimens was approved by the University’s ethics committee (approval code S-206/2005).

### 2.2. Tissue Microarray and Immunohistochemistry

On H&E-stained tissue slides of the GBC cohort, representative areas of invasive tumor and high-grade BilIN were marked by two pathologists. Tissue cores (1.0 mm diameter) of the marked regions were extracted from the donor blocks and embedded in duplicate into a new paraffin array block using a tissue microarrayer (TMA Grand Master Fa. Sysmex, Norderstedt, Germany). Immunohistochemistry was performed on an automated immunostainer (Ventana BenchMark Ultra, Ventana Medical Systems, Tucson, AZ, USA) using the biotin-free OptiView DAB IHC Detection Kit (Ventana Medical Systems, Oro Valley, AZ, USA). In brief, from the formalin fixed and paraffin-embedded TMA blocks 3 µm sections were cut, deparaffinized, rehydrated and pre-treated with an antigen retrieval buffer (Tris/Borat/EDTA, pH 8.4). For CD155 immunohistochemistry a citrate/acetate-based buffer was used (CC2, pH 6.5, Roche, Rotkreuz, Switzerland). After blocking of endogenous peroxidase, the slides were incubated with monoclonal antibodies directed against PD-L1 (clone SP263, 1.61 µg/mL, Roche), CD4 (clone SP35, 2.5 µg/mL, Roche), CD8 (clone SP57, 0.35 µg/mL, Roche) and PD-1 (clone NAT105, 2.21 µg/mL, Roche) at the provided dilutions of the ready-to-use-kits, followed by incubation with OptiView Universal Linker and OptiView HRP Multimer. For CD155 immunohistochemistry the clone D8A5G (Cell Signaling, Cambridge, UK) was used at a dilution of 1:200. Visualization was achieved using DAB-Chromogen. Before mounting, slides were counterstained with hematoxylin.

TIGIT immunohistochemistry was performed on another automated immunostainer (Autostainer Link 48, Agilent Technologies Inc., Santa Clara, CA, USA) using the Dako Real Detection System Peroxidase/AEC rabbit/mouse (Dako, Agilent Technologies Inc.) and antigen retrieval in citrate buffer (pH 6.0). The slides were incubated with a monoclonal antibody against TIGIT (clone TG1, OncoDianova, Hamburg, Germany) at a dilution of 1:100. Slides were developed using AEC-Chromogen and counterstained with hematoxylin.

For the assessment of major histocompatibility index-I (MHC-I), HER2 gene amplification status and microsatellite instability (MSI) see [22,23,24]. TMAs were digitalized at 400× magnification using a slide scanner (Aperio SC2, Leica Biosystems, Nussloch, Germany).

### 2.3. PD-L1 Evaluation

All sections were independently assessed by two pathologists with particular expertise in biliary tract cancer (BG and TA), blinded to clinical information. PD-L1 staining of the epithelial tumor component and of tumor-infiltrating lymphocytes in the stroma were analyzed separately and expressed as Tumor Proportion Score (TPS) and Immune Cell Score (IC), respectively, as defined before [25]. In addition, the Combined Positivity Score (CPS) was determined, taking into account both epithelial and immune cell staining [25].

All samples were confirmed to include at least 200 viable tumor cells. For the epithelial tumor component, in keeping with the current consensus on PD-L1 interpretation, only membranous staining was considered positive, irrespective of the staining intensity. Necroses were excluded from the analysis. For tumor infiltrating immune cells (TILs), both cytoplasmic and membranous staining were rated positive. Identification of immune cells was performed morphologically, taking into account H&E stained slides of the TMA. PD-L1 was evaluated at three cut-offs selected in agreement with previous therapeutic trials, as follows: TPS and IC (1%, 10% and 25%); CPS (scores 1, 10, 25). Ten cases of chronic cholecystitis served as negative control. Tonsil tissue was used as positive control.

### 2.4. Evaluation of CD4, CD8, PD-1, TIGIT and CD155

Immune cell densities were assessed semi-quantitatively on TMA slides taking the average of both representative tissue cores and expressed as number of positive cells/dot. Immunostainings for CD4, CD8, PD-1 and TIGIT were considered positive in the case of a specific cytoplasmic or membranous staining. CD155 immunohistochemistry was considered positive if a specific membranous signal was present and expressed relative to the number of the analyzed cells. Quantification was in all instances performed in conjunction with morphology to avoid false-positive results (e.g.: unspecific staining of necrosis, stroma cells, epithelium).

### 2.5. Statistical Analysis

Quantitative data are displayed as median with corresponding 25th and 75th percentiles (interquartile range). Differences in quantitative variables were compared using the Wilcoxon-Mann-Whitney test. Differences in frequencies were analyzed using the χ^2^ test or Fisher’s exact test, where appropriate. Survival times were graphed using the Kaplan-Meier method and differences assessed by the Mantel-Cox log rank test. Correlation analyses were performed using Pearson’s correlation test. For multivariate survival analysis, cox proportional hazards regression models were computed using XLSTAT Version 2020.3 (Addinsoft, Paris, France) followed by Wald’s test for assessing statistical significance. All other statistical analyses were performed with GraphPad Prism 6.0 (GraphPad Software, Inc., La Jolla, CA, USA). P-values below 0.05 were considered statistically significant.

## 3. Results

### 3.1. PD-L1 Expression in Gallbladder Cancer

**Figure 1:**
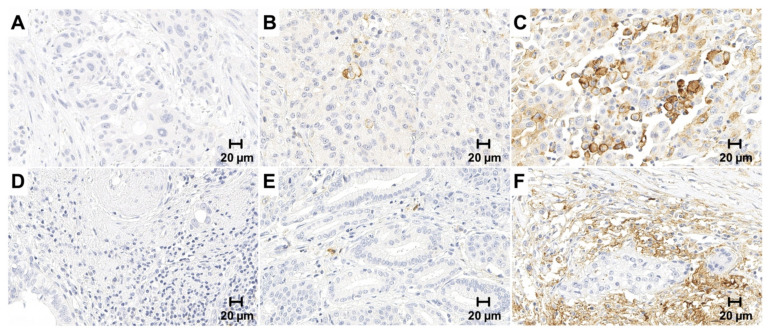
PD-L1 immunohistochemistry in gallbladder cancer and infiltrating immune cells. PD-L1 expression was evaluated on both the tumor and immune cells and expressed as Tumor Proportion Score (TPS), Immune Cell Score (IC) and Combined Positivity Score (CPS), respectively. Only membranous staining was counted. Upper panel: Representative microphotographs of negative (**A**) low (<10%); (**B**) high (>10%); (**C**) PD-L1 expression in the tumor cells. Lower Panel: Representative microphotographs of negative (**D**) low (<10%); (**E**) high (>10%); (**F**) PD-L1 expression in tumor infiltrating immune cells. Original magnification 400×.

Two of the 131 histologically confirmed GBC cases had to be excluded due to insufficient material that did not meet the evaluation criteria (129 out of 131, drop-out rate 1.5%). PD-L1 expression was assessed immunohistochemically using the SP263 clone both on tumor cells and infiltrating immune cells and reported as TPS, IC and CPS, respectively (Figure 1). The majority of GBC cases were negative for PD-L1 in the tumor cell compartment with only 14.7% of all cases exhibiting a TPS of at least 1% (Table 1). When applying higher cut-offs of 10% and 25% the number of TPS-positive GBC cases further decreased to 4.7% and 3.1%, respectively. Among the cases with any PD-L1 positivity in the tumor cells, median TPS equaled 5% while the maximum was 80%. PD-L1 expression was significantly more frequent in immune cells compared to tumor cells (*p* < 0.001) with an IC of at least 1% in one third of all cases (33.3%), while an IC above 10% and 25% was noted in 15% and 3% of the cases, respectively. Also, individual TPS and IC were significantly correlated (*p* < 0.0001, *r* = 0.67). All ten cholecystitis specimens included as controls were entirely negative for PD-L1 both in the epithelium and immune cells (Figure S1).

Since PD-L1 expression may be subject to intratumoral heterogeneity as reported in other malignancies [26,27], we repeated PD-L1 immunohistochemistry on all 19 samples with any tumoral PD-L1 positivity and an equal number of randomly selected negative samples to prove reproducibility of our methodology. None of the 19 negative samples by TMA analysis turned positive by evaluation of a whole slide specimen. In the 19 positive samples, expression quantities corresponded well to those determined in the TMA with a high level of agreement both with respect to the TPS (*r* = 0.97, *p* < 0.0001) and IC (*r* = 0.99, *p* < 0.0001) (Figure S2).

### 3.2. PD-L1 Expression in High-Grade Biliary Intraepithelial Neoplasia

**Figure 2:**
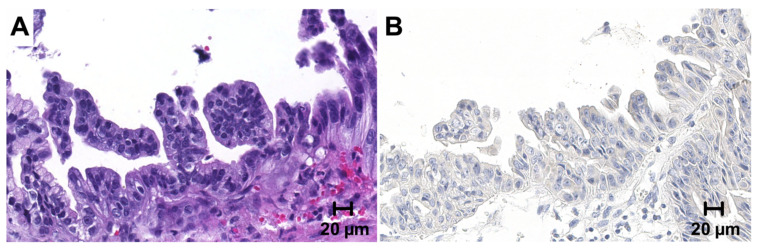
PD-L1 immunohistochemistry in high-grade biliary intraepithelial neoplasia. PD-L1 expression was also assessed in a number of 74 concomitant high-grade biliary intraepithelial neoplasia (BilIN) precursor lesions. (**A**) (HE). All BilIN lesions showed complete negativity for PD-L1 (**B**). Original magnification 400×.

Concomitant high-grade biliary intraepithelial lesions were available in a subset of 74 patients, of which two had to be excluded due to detachment of tissue cores. The remaining 72 cases uniformly lacked PD-L1 expression in the precursor epithelium (Figure 2). PD-L1 expression in surrounding immune cells could not be evaluated separately due to adjacent invasive tumor.

### 3.3. Correlation of PD-L1 Expression with Clinicopathological Criteria

To determine whether specific clinicopathological criteria might predict PD-L1 positivity, all scores were correlated with available histological and clinical information. Detailed clinicopathological characteristics of the entire cohort stratified for a 10% TPS cut-off point are displayed in Table 2. Stratified clinicopathological characteristics for all other cut-off values and scoring systems are listed in Tables S1–S8. Irrespective of the applied cut-off value, a high TPS was significantly correlated with poor tumor differentiation (*p* = 0.018, *p* = 0.024, *p* = 0.016 for TPS ≥ 1%, 10% and 25%, respectively; Fisher’s exact test). A similar, but less prominent association was also confirmed for a high CPS (Tables S7 and S8), but not for the IC. PD-L1 expression was highly variable depending on the histological subtype, in particular when using a high cut-off value (Table 3). At a TPS of ≥25%, only one of the four positive cases demonstrated conventional tumor histomorphology (i.e., ductal/tubular), while the remaining three were of a distinct subtype (adenosquamous, signet ring and solid). An association towards nontypical tumor morphologies was also detected when applying the IC and CPS. All cases with a papillary or intestinal morphology were entirely negative for PD-L1 expression in the tumor, irrespective of the applied cut-off value. Except for pT stage at a CPS cut-off of 1, no significant correlation with any other clinico-pathological parameter was observed with respect to all three scoring systems, including age, sex, UICC stage and lymph node or distant metastasis.

### 3.4. Correlation of PD-L1 Expression with MHC I Expression and HER2 Amplification

Due to its pivotal role for immune cell recognition, we correlated PD-L1 quantity in the tumor and TILs with expression data of MHC-I, which we already determined in a previous study [23]. However, no significant association was found either for the TPS (*p* = 0.4784, *r* = 0.36), IC (*p* = 0.46, *r* = 0.12) or CPS (*p* = 0.48, *r* = 0.11). Since studies postulated a significant crosstalk between HER2 and PD-1/PD-L1 signaling, we next correlated PD-L1 expression with HER2 gene amplification status that we assessed before [22,28]. PD-L1 expression was neither significantly correlated with HER2 gene amplification in the tumor (TPS, *p* = 0.69), nor in TILs (IC, *p* = 0.76) with three of the four HER2-positive cases completely lacking tumoral PD-L1 expression. Since none of the included samples exhibited microsatellite instability (MSI-H) as assessed previously [24], the general observation of enhanced PD-L1 expression in tumors with mismatch repair deficiency could not be substantiated in this study.

### 3.5. High Tumoral PD-L1 Expression Is Associated with Worse Survival

**Figure 3:**
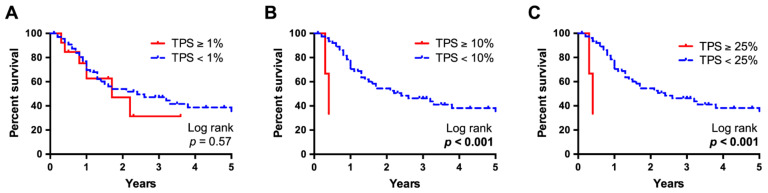
Kaplan-Meier survival analysis, stratified for Tumor Proportion Score. Survival data of a subset of 104 patients with available follow-up information were compared using Kaplan-Meier curves and log-rank testing. While at a Tumor Proportion Score (TPS) cut-off value at 1% no significant differences were observed (**A**), at a 10% (**B**) and 25% (**C**) cut-off, median survival was significantly worse in patients with high PD-L1 expression and six times lower than of those with lower expression (*p* < 0.001, 0.4 years vs. 2.2 years for both analyses).

Survival data were available for a subset of 104 patients. Kaplan-Meier survival curves were compared using log-rank testing, which revealed a significantly worse overall survival of those patients with a particularly high PD-L1 expression in the tumor cells (Figure 3). Median survival was six times lower in cases with a TPS above 10% and comparably poor in cases with a TPS above 25% (*p* = 0.0003, 0.4 years vs. 2.2 years, for both analyses). No significant difference was detected when using a minimal 1% TPS cutoff value (*p* = 0.567, 1.7 years vs. 2.4 years). PD-L1 expression in immune cells (IC) or tumor and immune cells (CPS) did not show a significant impact on survival, irrespective of the cut-off values (Figure S3). Multivariate analysis computed using a Cox proportional hazards regression model under inclusion of UICC staging revealed a high TPS (cutoff 10%) to be an independent negative prognosticator with an impact on survival almost twice as high as an advanced UICC stage in our cohort (*p* = 0.009, hazard ratio 9.4; Table 4).

### 3.6. PD-L1 Expression Is Associated with Increased CD4+-, CD8+- and PD-1+ Immune Cell Densities

**Figure 4:**
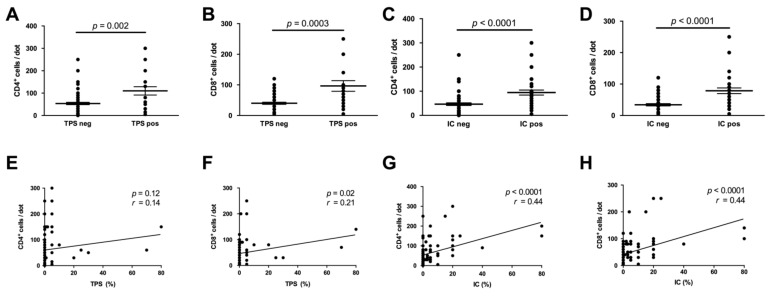
PD-L1 expression is associated with immune cell phenotype and density. Cases with positive PD-L1 expression either in tumor (Tumor Proportion Score (TPS), (**A**,**B**) or immune cells (Immune Cell Score) (IC), (**C**,**D**) displayed significantly increased intra- and peritumoral CD4^+^- and CD8^+^-immune cell densities. Correlation analysis confirmed this association, reaching significance for the correlation of the TPS with CD8^+^-cell density and for the correlation of the IC with CD4^+^-/CD8^+^-cell density (**E**–**H**).

To study the relationship between PD-L1 expression and host antitumor immunity in the microenvironment, we next characterized the tumor immune cell infiltrate by immunohistochemical quantification of CD4^+^- and CD8^+^ T-cells. Stratification for PD-L1 expression revealed that tumors with positive PD-L1 expression either on tumor or immune cells (TPS/IC ≥ 1%) showed significantly increased infiltration of CD4^+^- and CD8^+^ T-cells (*p* = 0.002/*p* = 0.0003 for TPS; *p* < 0.0001/*p* < 0.0001 for IC) (Figure 4A–D). The median number of CD8^+^ T-cells was nearly three times higher in tumors with positive tumoral PD-L1 expression as compared to tumors lacking PD-L1 expression (30 vs. 80 cells/dot). While the densities of both CD4^+^- and CD8^+^ T-cells positively correlated with the IC (each *p* < 0.0001), considering the TPS this correlation was only significant for CD8^+^ T-cells (*p* = 0.02) (Figure 4E–H).

**Figure 5:**
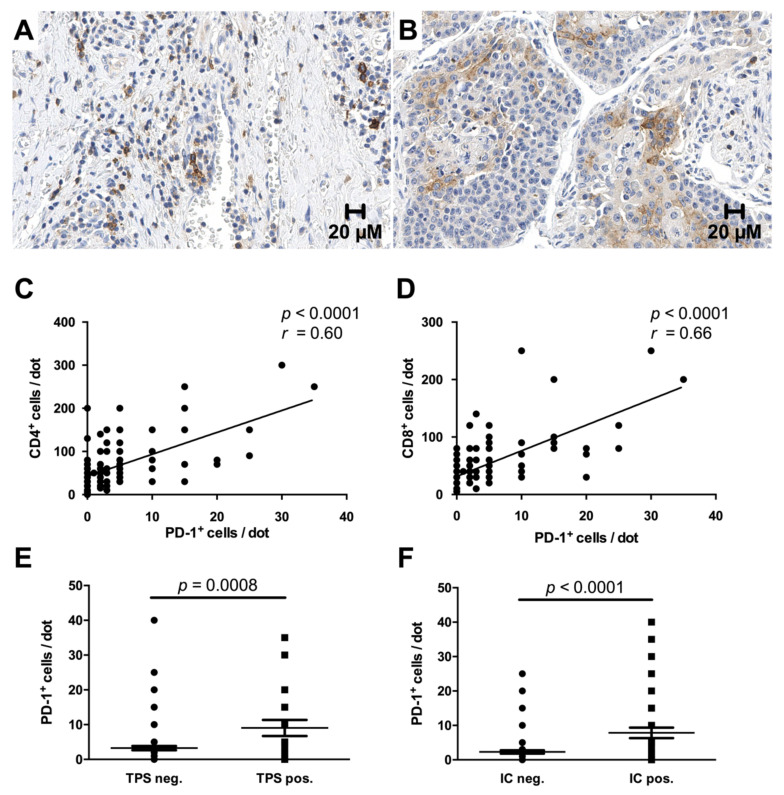
PD-1^+^ immune cells are increased in tumors with PD-L1 expression. More than half of the cases demonstrated strong membranous and cytoplasmic staining for PD-1 in a fraction of intra- and peritumoral immune cells (**A**), while positivity in the tumor epithelium was only detected in a single case with adenosquamous morphology (**B**). Co-expression of PD-1 and CD4 or CD8 was confirmed by correlation analysis (**C**,**D**). Furthermore, enhanced PD-1 expression was observed in cases with a Tumor Proportion Score (TPS) or Immune Cell score (IC) ≥ 1% (**E**,**F**). Original magnification in (**A**,**B**): 400×.

Immunoreactivity for PD-1 was detected in the immune cells in more than half of the cases (52.3%) with a mostly strong cytoplasmic and membranous signal (Figure 5A). Interestingly, clear immunoreactivity for PD-1 was also found in the tumor epithelium in one of the cases with adenosquamous morphology (Figure 5B). In all other cases, the tumor cells were entirely negative for PD-1. Morphologically evident co-expression of PD-1 and both CD4^+^ and CD8^+^ was confirmed by correlation analysis, which showed a more prominent association with CD8^+^ T-cells (*p* < 0.0001) (Figure 5C,D). Analogous to previous studies, PD-1 expression was significantly higher in cases with concomitant positivity for PD-L1 both in tumor and immune cells (*p* = 0.0008 for TPS, *p* < 0.0001 for IC) (Figure 5E,F) [29,30].

### 3.7. Expression of the Inhibitory Immune Receptor TIGIT and Its Ligand CD155

Recent studies identified the interaction of the immunomodulatory receptor TIGIT with its major ligand CD155 as a novel immune-checkpoint that may mediate tumor resistance to anti-PD-L1 immunotherapy [31,32]. To evaluate the potential of blockage of the TIGIT/CD155 axis for GBC, we quantified the expression levels on tumor cells and TILs by immunohistochemistry. We found TIGIT to be exclusively expressed by mononuclear immune cells with absent immunoreactivity in the tumor epithelium and a mostly granular cytoplasmic staining pattern.

**Figure 6:**
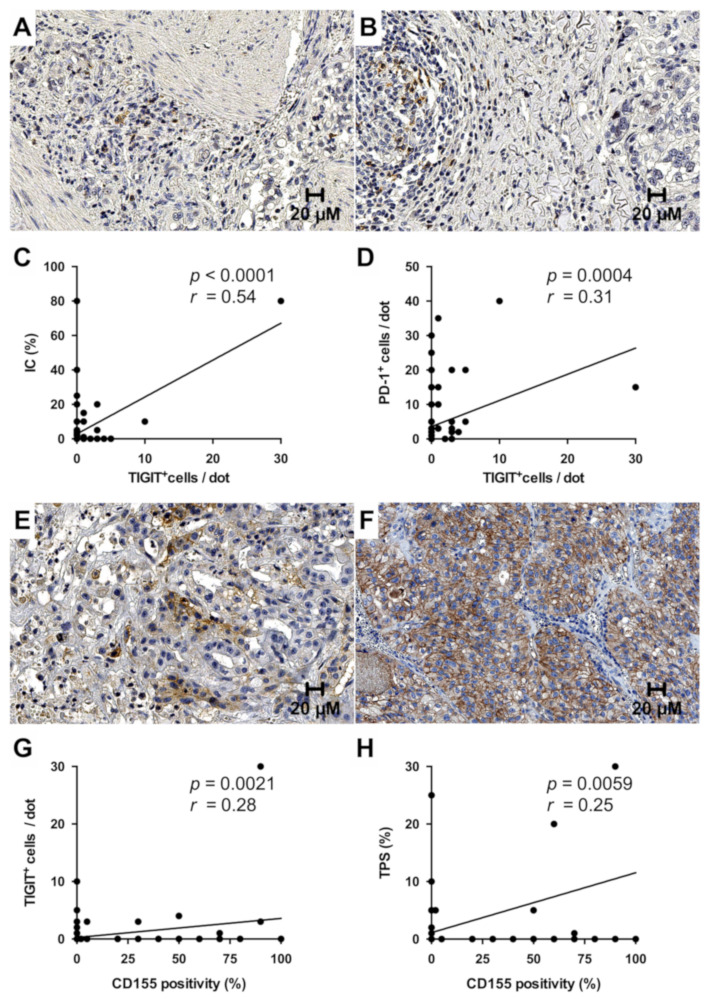
Expression of TIGIT and CD155 in gallbladder cancer. TIGIT was exclusively expressed by tumor-associated immune cells and mostly restricted to single lymphocytes (**A**). Only a few cases showed aggregated TIGIT^+^ immune cell clusters (**B**). TIGIT positivity in immune cells closely correlated with PD-L1 and PD-1 expression (**C**,**D**). CD155 immunohistochemistry showed a mostly strong membranous signal, that ranged from focal (**E**) to ubiquitous (**F**). CD155 positivity significantly correlated with the number of TIGIT^+^ immune cells and PD-L1 expression in tumor cells (**G**,**H**). Original magnification in (**A**,**B**,**E**,**F**): 400×.

While TIGIT-positive TILS were identified in 14.8% (19/128) of all evaluable cases, in the majority positivity was confined to single scattered immune cells (Figure 6A). Strikingly, the only case with a relatively high number of TIGIT-positive cells in the immune infiltrate (approx. 10% of the TILs) (Figure 6B) also displayed the highest IC. Correlation analysis confirmed co-expression of TIGIT and both PD-L1 as well as PD-1 in TILs (*p* < 0.0001 for IC, *p* = 0.0004 for PD-1), while no association with the TPS was detected (Figure 6C,D).

In contrast to TIGIT, CD155 was exclusively expressed by the tumor epithelium with a foremost moderate to strong membranous immunoreactivity. Positivity for CD155 was identified in 25.2% (30/119) of all evaluable cases, ranging from focal staining (Figure 6E) to ubiquitous immunoreactivity in all tumor cells (Figure 6F). Among the CD155-positive cases, median CD155 expression was 50%. Interestingly, tumoral CD155 positivity significantly correlated with the number of TIGIT-positive TILs (*p* = 0.002) and PD-L1 expression in tumor cells (*p* = 0.006 for TPS) (Figure 6G,H).

### 3.8. CD155 Is a Negative Prognosticator in Western-World Gallbladder Cancer

**Figure 7:**
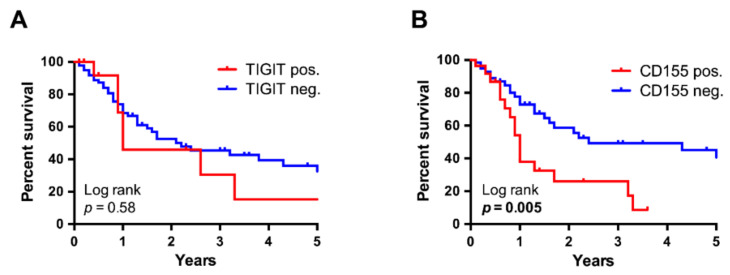
Kaplan-Meier survival analysis, stratified for TIGIT- and CD155 positivity. While the survival curves of cases with and without TIGIT^+^ immune cells followed a similar trend (*n* = 103) (**A**), CD155 positivity was associated with a significantly worse survival by log-rank testing (*n* = 95, *p* = 0.005, 1.0 vs. 2.4 years) (**B**).

Finally, we assessed whether TIGIT or CD155 expression may influence survival analogous to a high TPS. While TIGIT positivity in TILs was not associated with prognosis (*n* = 103, n = 14 (pos.) vs. n = 89 (neg.), *p* = 0.578) (Figure 7A), Kaplan-Meier analysis revealed CD155 to be a significant negative prognosticator with a median survival of only one year in the CD155-positive group compared to 2.4 years in those cases with absent CD155 expression (*n* = 95, n = 28 (pos.) vs. n = 67 (neg.), *p* = 0.005) (Figure 7B).

## 4. Discussion

Using stringent evaluation criteria and applying all current scoring systems, we herein assessed PD-L1 expression in a comprehensive and large, monocentric Western-world GBC cohort recruited at Heidelberg University Hospital. Our results reveal a frequency of approximately one out of seven cases to exhibit immunoreactivity for PD-L1 in the invasive tumor, of which roughly one third (5% of the whole cohort) showed a higher expression with a TPS of at least 10%. Interestingly, adjacent high-grade BilIN lesions uniformly lacked PD-L1 expression. In the TILs, PD-L1 expression was more frequent and significantly correlated with the individual TPS. High tumoral PD-L1 expression was significantly associated with high tumor grading, atypical morphological subtypes and markedly worse overall survival, despite the small number of positive cases and normalization for UICC stage. To date, no longitudinal studies exist that specifically address the outcome of PD-1/PD-L1 blockade in GBC. Current evidence is limited to several small sample size, single and multi-agent phase I and II studies on biliary tract cancer, that subsumed gallbladder cancer together with various subtypes of cholangiocarcinoma and the majority of which did not include predictive testing of PD-L1 expression prior to treatment initiation [33,34,35,36,37,38]. These studies report a heterogenous, yet promising partial response rate following PD-1/PD-L1 inhibition in a range from 3% to 37%. Follow-up data of the KN158 phase II trial as the largest study so far revealed a durable antitumor activity induced by pembrolizumab with a partial response in approximately 6% of all patients [33]. Only including patients with a tumoral PD-L1 expression of at least 1%, the preceding KN028 phase I trial showed an objective response rate of 13% [33]. A meta-analysis of the published trials on PD-1/PD-L1 inhibition in GBC is provided in Table 5. Additional evidence comes from single case reports: Kong et al. reported a significant response after combined radio- and immunotherapy with complete resolution of metastases in retroperitoneal lymph nodes and lungs in a patient with exceptionally high PD-L1 expression in 50% of the tumor cells [39]. Comparably favorable results were also observed by Li et al. in a patient with metastatic GBC and 10% tumoral PD-L1 expression, being still alive with controlled disease 14 months after diagnosis upon combined chemotherapy and pembrolizumab administration [40]. Though the predictive value of PD-L1 expression in GBC is still controversial, these studies suggest tumoral PD-L1 expression to serve as a potentially useful biomarker in GBC immunotherapy. PD-L1 expression is the most widely adopted parameter for selection of patients that may profit from immunotherapy [41]. Yet, to date only three studies conducted in Asia and India exist on the distribution of PD-L1 expression in GBC. In the largest study by Neyaz et al. [18], PD-L1 expression was seen in 23% of the tumor cells and 24% of TILs with 14.9% of the cases demonstrating a high tumoral expression of at least 10%. The remaining two studies correspondingly reported a somewhat lower tumor cell positivity in approximately 18% of the patients, which is more in line with the frequency determined in this study [19,20]. The discrepancy may be partially attributable to different antibody clones and immunohistochemistry platforms but may also mirror biological features resulting from ethnical and environmental differences between Western and Eastern GBC cohorts. In contrast to the study by Neyaz et al. [18], we identified a highly significant negative prognostic effect of a high tumoral PD-L1 expression above a cut-off value of 10%, which was associated with a six times lower median survival. This strong influence remained after inclusion of UICC stage in multivariate analysis, exceeding the negative prognostic value of a high UICC stage. However, this association vanished upon inclusion of patients with any PD-L1 positivity. On the assumption that the marked biological effect of a particularly high PD-L1 expression with regard to survival may to some extent also translate in responsiveness to its inhibition upon immunomodulation, the findings of this study may suggest a more restrictive PD-L1 based patient selection for inclusion in prospective clinical trials. Moreover, the uneven distribution of PD-L1 positivity across the morphological subtypes may help clinicians to consider PD-L1 testing and subsequent therapeutic immunomodulation as ultima ratio particularly in patients with rare subtypes. After initial restriction to tumoral PD-L1 expression in the first FDA-approved predictive PD-L1 companion diagnostics, more recently scoring algorithms evolved that implement PD-L1 expression in immune cells or a combination of both [17]. Accordingly, in this study we also assessed the IC and CPS, which in contrast to the TPS were not associated with survival. In keeping with previous studies, we identified a close inter-correlation between PD-L1 expression in tumor and immune cells [42,43]. The correlated expression patterns in tumor and immune cells as seen here fit with the current perception, that acquired PD-L1 upregulation seems to be driven by distinct anti-tumor immunity factors in the microenvironment, such as interferon gamma (IFN-γ), potentially acting on both compartments [44]. In addition to the different scoring systems, it has to be noted, that there are no uniform cut-off values for PD-L1 expression and thresholds for approved first- or second line immunotherapies vary a lot across different cancer entities. Here, significant associations were seen at comparably high TPS cut-off levels of 10% and 25%, indicating the necessity of a certain degree of positivity to translate into effective immune evasion.

Recently, we reported complete remission of metastasized GBC in a young patient, achieved by dual inhibition of molecularly proven amplified HER2 gene and determined a low frequency of HER2 gene amplification in GBC in a follow-up study [22,45]. In the current study, correlation analysis did not reveal a significant association between HER2 status and PD-L1 expression, indicating that HER2 gene amplification does not trigger PD-L1 upregulation in GBC. We also did not detect a significant relationship between MHC I and PD-L1 expression. Though a correlation seems reasonable given the common induction mechanisms (e.g., Interferon-γ), this result is in line with a previous study by Perea et al. [46] on a cohort of non-small cell lung cancer, in which no association between MHC-I and PD-L1 expression was detected. In contrast, in a study on serous ovarian cancer Aust et al. [47] found a negative correlation and suggested mutual exclusiveness, indicating that the crosstalk between MHC-I and PD-L1 is probably more complex and maybe tumor type-dependent. In line with previous studies on PD-L1 expression in cancer, we detected increased CD8^+^ and CD4^+^ T-cell densities in patients with high PD-L1 expression in tumor or immune cells of GBC [30,48]. This finding underscores the fundamental relevance of T-cells for immune evasion, mechanistically assumed to be the source PD-L1 expression via secretion of multiple cytokines [4].

Cancer development in the extrahepatic biliary tract is considered to follow a stepwise process through precursor lesions of increasing dysplasia [49]. Interestingly, we found PD-L1 to be completely absent in high-grade BilIN by immunohistochemistry. This might be attributed to the absence of distinct late occurring molecular events in BilIN. However, probably even more important, as opposed to GBC the epithelium in BilIN is not exposed to the various PD-L1 inducing cytokines within the tumor microenvironment, being surrounded by a still intact basal membrane.

A growing body of evidence documents that immune cell exhaustion upon chronic tumor antigen exposure is mediated by multiple inhibitory pathways [50,51]. Beyond PD-1/PD-L1, the TIGIT/CD155 axis has recently emerged as a putative new immune checkpoint that may be implicated in resistance to current immunotherapeutic agents [31,32]. TIGIT belongs to the Ig superfamily and impedes T-cell function by several mechanisms, of which binding to its major ligand CD155 seems to be of critical importance for humans [51]. The promising findings in preclinical studies have led to the recent launch of several phase I and II trials that evaluate anti-TIGIT therapy in solid tumors and yielded encouraging results in preliminary analyses [52,53]. Most notably, the phase II CITYSCAPE trial on locally advanced or metastatic non-small cell lung cancer demonstrated a nearly doubled overall response rate of combined anti-TIGIT/-PD-1 therapy compared to anti-PD-1 therapy alone [52]. Laying the foundation for analogous clinical trials in GBC, this is the first study that documents presence of the TIGIT/CD155 axis in GBC. While mostly scattered TIGIT^+^ immune cells were identified in approximately 15% of all cases, CD155 was roughly positive in one fourth of the cohort with some tumors even exhibiting a ubiquitously strong and universal expression pattern. The intercorrelations between PD-1, PD-L1, TIGIT and CD155 substantiate the phenomenon that tumors often co-express multiple inhibitory molecules for effective immune evasion [54,55].

Given the reported intratumoral heterogeneity of PD-L1 expression in other cancer forms [26,27], one possible limitation of this study is the usage of TMAs. To address this issue, we used two tissue cores from different tumor regions and performed an agreement analysis with a number of almost 40 corresponding whole slides. Though we expectedly detected some slight differences in single samples, the overall high rate of concordance demonstrated the general validity of our approach. Previous meta analyses confirmed this finding for all kinds of markers beyond PD-L1 with concordance of phenotypic expression patterns ranging from 80–100% [56,57]. In addition, TMA usage better mimics the clinical situation as PD-L1 testing is mostly being performed on small biopsies, given its typical indication for metastatic disease ineligible for surgical resection. Regarding the survival analysis, the small group sizes of four (cut-off 10% TPS) and six (cut-off 25% TPS) patients in the PD-L1 positive group limit the generalizability of this finding and require confirmation in future studies on preferably even larger cohorts.

## 5. Conclusions

In summary, in this study we show that PD-L1 is upregulated in tumor and immune cells in a subset of Western GBC in late tumorigenesis and provide evidence for TIGIT/CD155 axis as a new immune checkpoint for complement therapy in GBC. Given the marked negative prognostic value of tumor cell derived PD-L1 shown here and the promising effects of TIGIT/CD155 complement therapy demonstrated in other tumors, this study further substantiates the need for prospective testing of (combined) PD-L1- and TIGIT/CD155 inhibition in GBC and suggests to particular address patients with a high TPS.

## Figures and Tables

**Table 1:** Overview of PD-L1 expression in the cohort.

PD-L1	<1%	1–9%	10–24%	>25%
TPS	110 (85%)	13 (10%)	2 (2%)	4 (3%)
IC	86 (67%)	23 (18%)	16 (12%)	4 (3%)
CPS	84 (65%)	19 (15%)	13 (10%)	13 (10%)

Abbreviations: CPS, Combined Positivity Score; IC, Immune Cell Score; TPS, Tumor Proportion Score.

**Table 2:** Clinicopathological characteristics (at 10% TPS cutoff).

Characteristic	PD-L1 neg. (TPS < 10%)	PD-L1 pos. (TPS ≥ 10%)	*p*-Value
GBC patients	123 (95.3%)	6 (4.7%)	
Age (years)	72.9 (63.6–78.8)	78.55 (65.2–82.8)	0.42
Sex			
female	80 (35.0)	6 (100%)	0.18
male	43 (65.0%)	0 (0%)	
Histology			
NOS	61 (49.6%)	2 (33.3%)	* 0.046
mucinous	19 (15.4%)	0 (0%)	
adenosquamous	15 (12.2%)	1 (16.7%)	
signet ring	15 (12.2%)	1 (16.7%)	
papillary	10 (8.1%)	0 (0.0%)	
solid	1 (0.8%)	2 (33.3%)	
intestinal	2 (1.6%)	0 (0.0%)	
UICC			
1	0 (0%)	0 (0%)	* 0.59
2	15 (15.2%)	0 (0%)	
3	58 (58.6%)	6 (100%)	
4	26 (26.3%)	0 (0%)	
NA	24	0	
pT			
1	6 (4.9%)	0 (0%)	* 0.22
2	52 (42.3%)	1 (16.7%)	
3	57 (46.3%)	5 (83.3%)	
4	8 (6.5%)	0 (0%)	
pN			
0	27 (42.9%)	1 (50.0%)	0.98
1	36 (57.1%)	1 (50.0%)	
X	60	4	
pM			
1	22 (17.9%)	0 (0%)	0.59
X	101 (82.1%)	6 (100%)	
L			
0	47 (38.2%)	1 (16.7%)	0.41
1	76 (61.8%)	5 (83.3%)	
V			
0	57 (46.3%)	2 (33.3%)	0.69
1	66 (53.7%)	4 (66.7%)	
Pn			
0	63 (51.2%)	2 (33.3%)	0.68
1	60 (48.8%)	4 (66.7%)	
R			
0	46 (46.5%)	3 (60.0%)	* 0.66
1	47 (47.5%)	2 (40.0%)1	
2	6 (6.1%)	0 (0%)	
X	24	1	
Grading			
G1	5 (4.1%)	0 (0%)	* 0.024
G2	76 (61.8%)	1 (16.7%)	
G3	42 (34.1%)	5 (83.3%)	

Fisher’s exact test, χ^2^ test or Mann-Whitney test (age) were used. * denotes NOS + mucinous + papillary + intestinal vs. adenosquamous + signet ring + solid; UICC 1 + 2 vs. 3 + 4; pT1 + 2 vs. 3 + 4; R0 vs. R1 + 2; G1 + 2 vs. G3. Abbreviations: GBC, gallbladder cancer; UICC, Union for International Cancer Control.

**Table 3:** Distribution of tumor morphologies, stratified for Tumor Proportion Score.

TPS	<10%	≥10%
NOS	97%	3%
Mucinous	100%	0%
Adenosquamous	94%	6%
Signet ring	94%	6%
Papillary	100%	0%
Solid	33%	67%
Intestinal	100%	0%

Abbreviations: NOS, not otherwise specified; TPS, Tumor Proportion Score.

**Table 4:** Multivariate survival analysis (Cox regression model).

Variable	Groups	HR	Lower CI (95%)	Upper CI (95%)	*p*-Value
TPS	TPS < 10%	1	-	-	-
	TPS ≥ 10%	9.404	1.733	51.020	0.009
UICC stage	UICC 2	1	-	-	-
	UICC 3	4.649	1.320	16.372	0.017
	UICC 4	5.469	1.468	20.375	0.011

Abbreviations: CI, confidence interval; HR, hazard ratio; TPS, Tumor Proportion Score; UICC, Union for International Cancer Control. Reported *p*-values were calculated using Wald’s test.

**Table 5:** Meta-analysis of trials on PD-1/PD-L1 inhibition in gallbladder cancer.

Authors [Reference]	Country	Study Design	Agent	No of Patients	No of GBC Patients	Outcome
Bang et al. [33]	Japan	Phase Ib	Pembrolizumab	24	-	13% PR, mPFS 1.8 mos, mOS 6.2 mos.
Bang et al. [33]	Japan	Phase II	Pembrolizumab	104	-	5.8% PR, mPFS 2.0 mos, mOS 7.4 mos.
Ueno et al. [34]	Japan	Phase I	Nivolumab + cisplatin + gemcitabine	30	10	36.7% PR, mPFS 4.2 mos., mOS 15.4 mos.
Ueno et al. [34]	Japan	Phase II	Nivolumab	30	10	3% PR, mPFS 1.4 mos., mOS 5.2 mos
Kim et al. [38]	USA	Phase II	Nivolumab	54	14	22% PR, mPFS 3.98 mos., mOS 14.22 mos.
Ioka et al. [35]	Japan	Phase I	Durvalumab + tremelimumab	65	16	10.8 % PR, mPFS 1.6 mos., mOS 10.1 mos.
Ioka et al. [35]	Japan	Phase II	Durvalumab	42	19	4.8 % PR, mPFS 1.5 mos., mOS 8.1 mos.
Fujiwara et al. [37]	Japan	Phase I	M7824 (MSB0011359C)	30	12	25% PR *
Yoo et al. [36]	USA	Phase I	Tremelimumab + RFA	16	2	12.5% PR, mPFS 3.4 mos., mOS 6.0 mos.

*: Outcome reported specifically for the GBC subcohort. Abbreviations: GBC, gallbladder cancer; mos, months; mOS, mean overall survival; mPFS, median progression-free survival; No, number; PR, partial response; RFA, radiofrequency ablation.

## Data Availability

Additional datasets analyzed during the current study are available from the corresponding author upon reasonable request.

## Supplementary Materials

The following are available online at https://www.mdpi.com/article/10.3390/cancers13071682/s1: Figure S1: PD-L1 expression in chronic cholecystitis, Figure S2: Correlation analysis of PD-L1 expression on tissue microarrays and whole slides, Figure S3: Kaplan-Meier survival analysis, stratified for Immune Cell Score and Combined Positivity Score. Table S1: Clinicopathological characteristics (at 1% TPS cutoff), Table S2: Clinicopathological characteristics (at 25% TPS cutoff), Table S3: Clinicopathological characteristics (at 1% IC cutoff), Table S4: Clinicopathological characteristics (at 10% IC cutoff), Table S5: Clinicopathological characteristics (at 25% IC cutoff), Table S6: Clinicopathological characteristics (at 1 CPS cutoff), Table S7: Clinicopathological characteristics (at 10 CPS cutoff), Table S8: Clinicopathological characteristics (at 25 CPS cutoff).

## Project Number

The study is registered under project #3225 of the Tissue Bank of the National Center for Tumor Diseases (NCT, Heidelberg, Germany).
