# Supplementary material for: Programmed Death Ligand-1 (PD-L1) Is an Independent Negative Prognosticator in Western-World Gallbladder Cancer
Source: Cancers (Basel). 2021 Apr 2;13(7):1682. doi: 10.3390/cancers13071682 (PMC8038183; doi:10.3390/cancers13071682)
Supplement: Supplementary file 1 [file cancers-13-01682-s001.pdf]

cancers

MDPI

Supplementary Material

Programmed death ligand-1 (PD-L1) is an independent negative prognosticator in Western-world gallbladder cancer

Thomas Albrecht et. al.

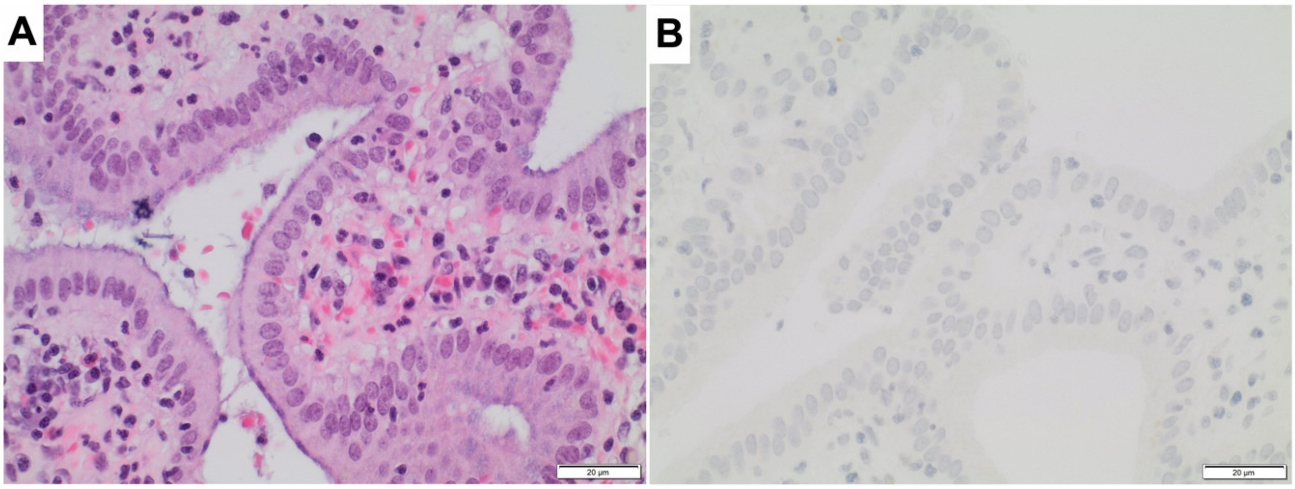

Figure S1. PD-L1 expression in chronic cholecystitis. Representative microphotographs of chronic cholecystitis specimens (A) (HE), which were uniformly negative for PD-L1 expression by immunohistochemistry (B), both in the epithelium and immune cells. Original magnification 400X.

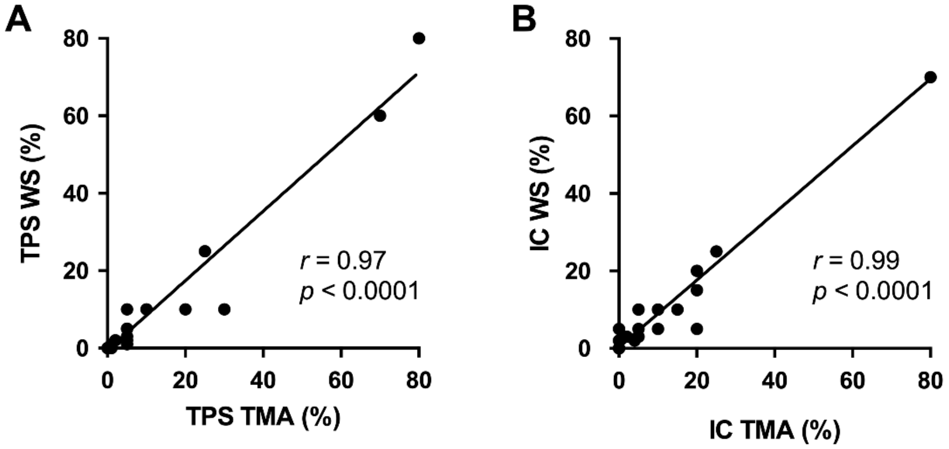

Figure S2. Correlation analysis of PD-L1 on tissue microarrays and whole slides. PD-L1 immunohistochemistry was repeated on whole slides of all 19 samples with tumoral PD-L1 positivity and an equal number of randomly selected negative samples. Correlation analysis of both the Tumor Proportion Score (TPS) (A) and Immune Cell Score (IC) (B) assessed on the tissue microarray with the results of corresponding whole slide (WS) revealed high agreement of both approaches.

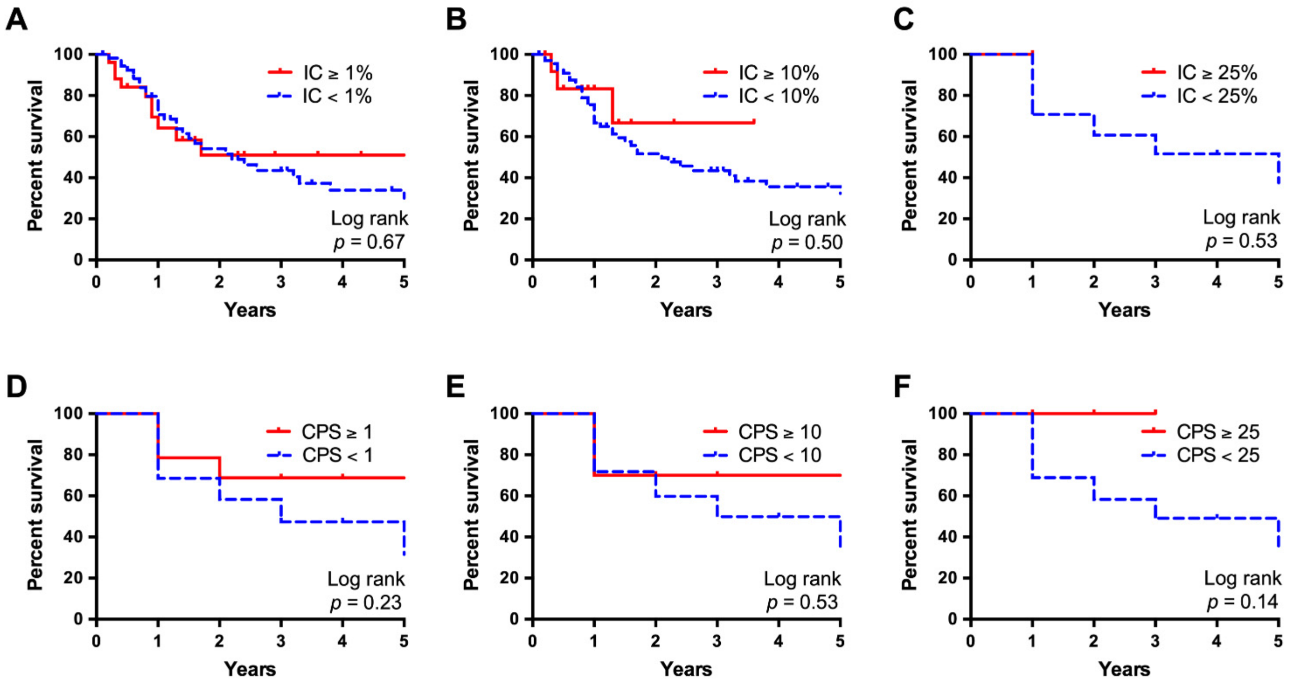

Figure S3. Kaplan-Meier survival analysis, stratified for Immune Cell Score and Combined Positivity Score. Irrespective of the cut-off point, no significant differences in survival were detected both for the Immune Cell Score (IC) and Combined Positivity Score (CPS). Upper panel: Survival curves for IC cut-off points at 1% (A), 10% (B) and 25% (C). Lower panel: Survival curves for CPS cut-off points at 1 (D), 10 (E) and 25 (F). *P*-values were calculated using the log-rank test.

Table S1. Clinicopathological characteristics (at 1% TPS cutoff).

Characteristic	PD-L1 neg. (<1%)	PD-L1 pos. (≥1%)	*p*-Value
GBC patients	110 (85.3%)	19 (14.7%)	
Age (years)	72.6 (63.6–79.1)	75.8 (64.1–80.4)	0.67
Sex			
Female	39 (35.5)	4 (21.1%)	0.30
Male	71 (64.5%)	15 (78.9%)	
Histology			
NOS	52 (47.3%)	11 (57.9%)	
Mucinous	18 (16.4%)	1 (5.3%)	
Adenosquamous	13 (11.8%)	3 (15.8%)	
Signet ring	14 (12.7%)	2 (10.5%)	*0.40
Papillary	10 (9.1%)	0 (0%)	
Solid	1 (0.9%)	2 (10.5%)	
Intestinal	2 (1.8%)	0 (0%)	
UICC			
1	0 (0%)	0 (0%)	
2	14 (15.9%)	1 (5.9%)	
3	51 (58.0%)	13 (76.5%)	*0.46
4	23 (26.1%)	3 (17.6%)	
NA	22	2	
pT			
1	5 (4.5%)	1 (5.3%)	
2	49 (44.5 %)	4 (21.0%)	*0.08
3	49 (44.5%)	13 (68.4%)	

4	7 (6.5%)	1 (5.3%)	
pN			
0	26 (46.4%)	2 (20.0%)	
1	30 (53.6%)	8 (80.0%)	0.34
X	54	9	
pM			
1	20 (18.2%)	2 (10.5%)	0.53
X	90 (81.8%)	17 (89.5%)	
L			
0	40 (36.4%)	8 (42.1%)	0.62
1	70 (63.6%)	11 (57.9%)	
V			
0	49 (44.5%)	10 (52.6%)	0.62
1	61 (55.5%)	9 (47.4%)	
Pn			
0	56 (50.9%)	11 (57.9%)	0.63
1	54 (49.1%)	8 (42.1%)	
R			
0	40 (45.4%)	9 (56.2%)	
1	42 (47.7%)	7 (43.8%)	*0.59
2	6 (6.8%)	0 (0%)	
X	22	3	
Grading			
G1	5 (4.5%)	0 (0%)	
G2	70 (63.6%)	7 (36.8%)	***0.018**
G3	35 (31.8%)	12 (63.2%)	

Fisher's exact test, $\chi 2$ test or Mann-Whitney test (age) were used. *denotes NOS+mucinous+papillary+intestinal vs. adenosquamous+signet ring+solid; UICC 1 + 2 vs. 3 + 4; pT1+2 vs. 3 + 4; R0 vs. R1 + 2; G1 + 2 vs. G3. Abbreviations: GBC, gallbladder cancer; UICC, Union for International Cancer Control.

Table S2. Clinicopathological characteristics (at 25% TPS cutoff).

**Characteristic**	**PD-L1 neg. (TPS < 25%)**	**PD-L1 pos. (TPS ≥ 25%)**	***p*-value**
GBC patients	125 (96.9%)	4 (3.1%)	
Age (years)	72.9 (63.7–80.0)	71.8 (61.6–80.8)	0.89
Sex			
Female	82 (65.6%)	4 (100%)	0.30
Male	43 (34.4%)	0 (0%)	
Histology			
NOS	62 (49.6%)	1 (25.0%)	
Mucinous	19 (15.2%)	0 (0%)	
Adenosquamous	15 (12.0%)	1 (25.0%)	
Signet ring	15 (12.0%)	1 (25.0%)	*0.06
Papillary	10 (8.0%)	0 (0%)	
Solid	2 (1.6%)	1 (25.0%)	
Intestinal	2 (1.6%)	0 (0%)	

UICC			
1	0 (0%)	0 (0%)	
2	15 (14.9%)	0 (0%)	
3	60 (59.4%)	4 (100%)	*1.00
4	26 (25.7%)	0 (0%)	
NA	24	0	
pT			
1	6 (4.8%)	0 (0%)	
2	52 (41.6 %)	1 (25.0%)	*0.62
3	59 (47.2%)	3 (75.0%)	
4	8 (6.4%)	0 (0%)	
pN			
0	27 (42.9%)	0 (0%)	
1	36 (57.1%)	3 (100%)	0.34
X	62	1	
pM			
1	22 (17.6%)	0 (0%)	1.00
X	103 (82.4%)	4 (100%)	
L			
0	48 (38.4%)	0 (0%)	0.30
1	77 (61.6%)	4 (100%)	
V			
0	59 (47.2%)	1 (25.0%)	0.62
1	66 (52.8%)	3 (75.0%)	
Pn			
0	64 (51.2%)	3 (75.0%)	0.62
1	61 (48.8%)	1 (25.0%)	
R			
0	46 (46.0%)	3 (75.0%)	
1	48 (48.0%)	1 (25.0%)	*0.34
2	6 (6.0%%)	0 (0%)	
X	25	0	
Grading			
G1	5 (4.0%)	0 (0%)	
G2	77 (61.5%)	0 (0%)	***0.016**
G3	43 (34.4%)	4 (100%)	

Fisher's exact test, $\chi 2$ test or Mann-Whitney test (age) were used. *denotes NOS+mucinous+papillary+intestinal vs. adenosquamous+signet ring+solid; UICC 1+2 vs. 3+4; pT1+2 vs. 3+4; R0 vs. R1+2; G1+2 vs. G3. Abbreviations: GBC, gallbladder cancer; UICC, Union for International Cancer Control.

Table S3. Clinicopathological characteristics (at 1% IC cutoff).

**Characteristic**	**PD-L1 neg. (IC < 1%)**	**PD-L1 pos. (IC ≥ 1%)**	***p*-value**
GBC patients	86 (66.7%)	43 (33.3%)	
Age (years)	72.9 (63.5–81.7)	73.40 (64.7–78.2)	0.80
Sex			
Female	56 (65.1%)	30 (69.8%)	0.69
M1ale	30 (34.9%)	13 (30.2%)	

Histology			
NOS	43 (50.0%)	20 (46.5%)	***0.035**
Mucinous	15 (17.4%)	4 (9.3%)	
Adenosquamous	7 (8.1%)	9 (20.9%)	
Signet ring	10 (11.6%)	6 (14.0%)	
Papillary	8 (9.3%)	2 (4.7%)	
Solid	1 (1.2%)	2 (4.7%)	
Intestinal	2 (2.4%)	0 (0%)	
UICC			
1	0 (0%)	0 (0%)	*0.25
2	12 (17.9%)	3 (7.9%)	
3	41 (61.2%)	23 (60.5%)	
4	14 (20.9%)	12 (31.6%)	
NA	19	5	
pT			
1	5 (5.8%)	1 (2.3%)	*0.09
2	39 (45.3%)	14 (32.6%)	
3	39 (45.3%)	23 (53.5%)	
4	3 (3.5%)	5 (11.6%)	
pN			
0	21 (47.7%)	6 (27.3%)	0.28
1	23 (52.3%)	16 (72.7%)	
X	42	21	
pM			
1	14 (16.3%)	8 (18.6%)	0.81
X	72 (83.7%)	35 (81.4%)	
L			
0	29 (33.7%)	19 (44.2%)	0.25
1	57 (66.3%)	24 (55.8%)	
V			
0	37 (43.0%)	22 (51.2%)	0.45
1	49 (57.0%)	21 (48.8%)	
Pn			
0	44 (51.2%)	23 (53.5%)	0.85
1	42 (48.8%)	20 (46.5%)	
R			
0	30 (44.1%)	19 (52.8%)	*0.42
1	33 (48.5%)	16 (44.4%)	
2	5 (7.4%)	1 (2.8%)	
X	18	7	
Grading			
G1	3 (3.5%)	2 (4.7%)	*0.12
G2	56 (65.1%)	21 (48.8%)	
G3	27 (31.4%)	20 (46.5%)	

Fisher's exact test, $\chi 2$ test or Mann-Whitney test (age) were used. *denotes NOS+mucinous+papillary+intestinal vs. adenosquamous+signet ring+solid; UICC 1 + 2 vs. 3 + 4; pT1 + 2 vs. 3 + 4; R0 vs. R1 + 2; G1 + 2 vs. G3. Abbreviations: GBC, gallbladder cancer; UICC, Union for International Cancer Control.

Table S4. Clinicopathological characteristics (at 10% IC cutoff).

Characteristic	PD-L1 neg. (IC < 10%)	PD-L1 pos. (IC ≥ 10%)	*p*-value
GBC patients	109 (84.5%)	20 (15.5%)	
Age (years)	72.6 (63.6–80.0)	75.60 (64.6–80.0)	0.62
Sex			
Female	73 (67.0%)	13 (65.0%)	1.00
Male	36 (33.0%)	7 (35.0%)	
Histology			
NOS	53 (48.6%)	10 (50.0%)	
Mucinous	17 (15.6%)	2 (10.0%)	
Mdenosquamous	11 (10.1%)	5 (25.0%)	*0.18
Signet ring	14 (12.8%)	2 (10.0%)	
Papillary	10 (9.2%)	0 (0.0%)	
Dolid	2 (1.8%)	1 (5.0%)	
Intestinal	2 (1.8%)	0 (0.0%)	
UICC			
1	0 (0%)	0 (0%)	
2	14 (16.3%)	1 (5.3%)	
3	49 (57.0%)	15 (78.9%)	*0.30
4	23 (26.7%)	3 (15.8%)	
NA	23	1	
pT			
1	6 (5.5%)	0 (0%)	
2	46 (42.2 %)	7 (35.0%)	*0.34
3	50 (45.9%)	12 (60.0%)	
4	7 (6.4%)	1 (5.0%)	
pN			
0	25 (45.5%)	2 (18.2%)	
1	30 (55.5%)	9 (81.8%)	0.24
X	54	9	
pM			
1	20 (18.3%)	2 (10.0%)	0.52
X	89 (81.7%)	18 (90.0%)	
L			
0	38 (34.9%)	10 (50.0%)	0.22
1	71 (65.1%)	10 (50.0%)	
V			
0	47 (43.1%)	12 (60.0%)	0.22
1	62 (56.9%)	8 (40.0%)	
Pn			
0	56 (51.4%)	11 (55%)	0.81
1	53 (48.6%)	9 (45%)	
R			
0	39 (44.8%)	10 (58.8%)	
1	42 (48.3%)	7 (41.2%)	*0.30
2	6 (6.9%)	0 (0%)	
X	22	3	

Grading			
G1	4 (3.7%)	1 (5%)	
G2	68 (62.4%)	9 (45.0%)	*0.21
G3	37 (33.9%)	10 (50.0%)	

Fisher's exact test, $\chi 2$ test or Mann-Whitney test (age) were used. *denotes NOS+mucinous+papillary+intestinal vs. adenosquamous+signet ring+solid; UICC 1 + 2 vs. 3 + 4; pT1 + 2 vs. 3 + 4; R0 vs. R1 + 2; G1 + 2 vs. G3. Abbreviations: GBC, gallbladder cancer; UICC, Union for International Cancer Control.

Table S5. Clinicopathological characteristics (at 25% IC cutoff).

Characteristic	PD-L1 neg. (IC < 25%)	PD-L1 pos. (IC ≥ 25%)	*p*-value
GBC patients	125 (96.9%)	4 (3.1%)	
Age (years)	73.0 (63.9–80.2)	64.2 (51.5–72.2)	0.12
Sex			
Female	83 (66.4%)	3 (75.0%)	1.00
Male	42 (33.6%)	1 (25.0%)	
Histology			
NOS	63 (50.4%)	0 (0%)	
Mucinous	19 (15.2%)	0 (0%)	
Adenosquamous	14 (11.2%)	3 (75.0%)	
Signet ring	14 (11.2%)	1 (25.0%)	***0.005**
Papillary	10 (8.0%)	0 (0.0%)	
Solid	3 (2.4%)	0 (0.0%)	
Intestinal	2 (1.6%)	0 (0.0%)	
UICC			
1	0 (0%)	0 (0%)	
2	15 (14.9%)	0 (0%)	
3	61 (60.4%)	3 (75.0%)	*1.00
4	25 (24.8%)	1 (25.0%)	
NA	24	0	
pT			
1	6 (4.8%)	0 (0%)	
2	52 (41.6 %)	1 (25.0%)	*0.62
3	59 (47.2%)	3 (75.0%)	
4	8 (6.4%)	0 (0%)	
pN			
0	26 (41.3%)	1 (33.3%)	
1	37 (58.7%)	2 (66.7%)	0.96
X	62	1	
pM			
1	21 (16.8%)	1 (25.0%)	0.53
X	104 (83.2%)	3 (75.0%)	
L			
0	46 (36.8%)	2 (50.0%)	0.63
1	79 (63.2%)	2 (50.0%)	
V			
0	57 (45.6%)	2 (50.0%)	1.00
1	68 (54.4%)	2 (50.0%)	

Pn			
0	65 (52.0%)	2 (50.0%)	1.00
1	60 (48.0%)	2 (50.0%)	
R			
0	47 (47.0%)	2 (50.0%)	*1.00
1	47 (47.0%)	2 (50.0%)	
2	6 (6.0%)	0 (0%)	
X	25	0	
Grading			
G1	5 (4.0%)	0 (0%)	*0.14
G2	76 (60.8%)	1 (25.0%)	
G3	44 (35.2%)	3 (75.0%)	

Fisher's exact test, χ2 test or Mann-Whitney test (age) were used. *denotes NOS+mucinous+papillary+intestinal vs. adenosquamous+signet ring+solid; UICC 1 + 2 vs. 3 + 4; pT1+2 vs. 3 + 4; R0 vs. R1 + 2; G1 + 2 vs. G3. Abbreviations: GBC, gallbladder cancer; UICC, Union for International Cancer Control.

Table S6. Clinicopathological characteristics (at 1 CPS cutoff).

Characteristic	PD-L1 neg. (CPS< 1)	PD-L1 pos. (CPS ≥ 1)	*p*-value
GBC patients	84 (65.1%)	45 (34.9%)	
Age (years)	72.9 (63.4–81.4)	73.40 (65.3–78.3)	0.88
Sex			
Female	54 (64.3%)	32 (71.1%)	0.56
Male	30 (35.7%)	13 (28.9%)	
Histology			
NOS	41 (48.8%)	22 (48.9%)	*0.06
Mucinous	15 (17.9%)	4 (8.9%)	
Adenosquamous	7 (8.3%)	9 (20.0%)	
Signet ring	10 (11.9%)	6 (13.3%)	
Papillary	8 (9.5%)	2 (4.4%)	
Solid	1 (1.2%)	2 (4.4%)	
Intestinal	2 (2.4%)	0 (0.0%)	
UICC			
1	0 (0%)	0 (0%)	*0.16
2	12 (18.5%)	3 (7.5%)	
3	40 (61.5%)	24 (60.0%)	
4	13 (20.0%)	13 (32.5%)	
NA	19	5	
pT			
1	5 (6.0%)	1 (2.2%)	***0.043**
2	39 (46.4%)	14 (31.1%)	
3	37 (44.0%)	25 (55.6%)	
4	3 (3.6%)	5 (11.1%)	
pN			
0	21 (50.0%)	6 (25.0%)	0.14
1	21 (50.0%)	18 (75.0%)	
X	42	21	
pM			
1	13 (15.5%)	9 (20.0%)	0.62
X	71 (84.5%)	36 (80.0%)	

L			
0	29 (34.5%)	19 (42.2%)	0.45
1	55 (65.6%)	26 (57.8%)	
V			
0	37 (44.0%)	22 (48.9%)	0.71
1	47 (56.0%)	23 (51.1%)	
Pn			
0	44 (52.4%)	23 (51.1%)	1.00
1	40 (47.6%)	22 (48.9%)	
R			
0	29 (43.9%)	20 (52.6%)	*0.42
1	32 (48.5%)	17 (44.7%)	
2	5 (7.6%)	1 (2.6%)	
X	18	7	
Grading			
G1	3 (3.6%)	2 (4.4%)	*0.09
G2	55 (65.5%)	22 (48.9%)	
G3	26 (30.9%)	21 (46.7%)	

Fisher's exact test, $\chi 2$ test or Mann-Whitney test (age) were used. *denotes NOS+mucinous+papillary+intestinal vs. adenosquamous+signet ring+solid; UICC 1 + 2 vs. 3 + 4; pT1 + 2 vs. 3 + 4; R0 vs. R1 + 2; G1 + 2 vs. G3. Abbreviations: GBC, gallbladder cancer; UICC, Union for International Cancer Control.

Table S7. Clinicopathological characteristics (at 10 CPS cutoff).

Characteristic	PD-L1 neg. (CPS < 10)	PD-L1 pos. (CPS ≥ 10)	*p*-value
GBC patients	103 (79.8%)	26 (20.2%)	
Age (years)	72.6 (63.6–80.0)	75.60 (63.5–80.1)	0.79
Sex			
Female	68 (66.0%)	18 (69.2%)	0.82
Male	35 (34.0%)	8 (30.8%)	
Histology			
NOS	50 (48.5%)	13 (50.0%)	*0.22
Mucinous	17 (16.5%)	2 (7.7%)	
Adenosquamous	10 (9.7%)	6 (23.1%)	
Signet ring	14 (13.6%)	2 (7.7%)	
Papillary	9 (8.7%)	1 (3.8%)	
Solid	1 (1.0%)	2 (7.7%)	
Intestinal	2 (2.0%)	0 (0%)	
UICC			
1	0 (0%)	0 (0%)	*0.51
2	13 (16.0%)	2 (8.3%)	
3	45 (55.6%)	19 (79.2%)	
4	23 (28.4%)	3 (12.5%)	
NA	22	2	
pT			
1	5 (4.9%)	1 (3.8%)	*0.51
2	44 (42.7%)	9 (34.6%)	
3	47 (45.6%)	15 (57.7%)	
4	7 (6.8%)	1 (3.8%)	

pN			
0	24 (45.3%)	3 (23.1%)	
1	29 (54.7%)	10 (76.9%)	0.34
X	50	13	
pM			
1	20 (19.4%)	2 (7.7%)	0.24
X	83 (80.6%)	24 (92.3%)	
L			
0	35 (34.0%)	13 (50.0%)	0.17
1	68 (66.0%)	13 (50.0%)	
V			
0	44 (42.7%)	15 (57.7%)	0.19
1	59 (57.3%)	11 (42.3%)	
Pn			
0	53 (51.5%)	14 (53.8%)	1.00
1	50 (48.5%)	12 (46.2%)	
R			
0	36 (44.4%)	13 (56.5%)	
1	39 (48.1%)	10 (43.5%)	*0.35
2	6 (7.4%)	0 (0%)	
X	22	3	
Grading			
G1	3 (2.9%)	2 (7.7%)	
G2	67 (65.0%)	10 (38.5%)	*0.07
G3	33 (32.1%)	14 (53.8%)	

Fisher's exact test, $\chi 2$ test or Mann-Whitney test (age) were used. *denotes NOS+mucinous+papillary+intestinal vs. adenosquamous+signet ring+solid; UICC 1+2 vs. 3+4; pT1+2 vs. 3+4; R0 vs. R1+2; G1+2 vs. G3. Abbreviations: GBC, gallbladder cancer; UICC, Union for International Cancer Control.

Table S8. Clinicopathological characteristics (at 25 CPS cutoff).

Characteristic	PD-L1 neg. (CPS < 25)	PD-L1 pos. (CPS ≥ 25)	p-value
GBC patients	116 (89.9%)	13 (10.1%)	
Age (years)	72.9 (63.6–79.7)	73.40 (62.–80.5)	0.87
Sex			
Female	76 (65.5%)	10 (76.9%)	0.54
Male	40 (34.5%)	3 (23.1%)	
Histology			
NOS	58 (50.0%)	5 (38.5%)	
Mucinous	18 (15.5%)	1 (7.7%)	
Adenosquamous	13 (11.2%)	3 (23.1%)	***0.042**
Signet ring	14 (12.1%)	2 (15.4%)	
Papillary	10 (8.6%)	0 (0%)	
Solid	1 (0.9%)	2 (15.4%)	
Intestinal	2 (1.7%)	0 (0.0%)	

UICC			
1	0 (0%)	0 (0%)	
2	15 (16.1%)	0 (0%)	
3	53 (57.0%)	11 (91.7%)	*0.21
4	25 (26.9%)	1 (8.3%)	
NA	23	1	
pT			
1	6 (5.2%)	0 (0%)	
2	49 (42.2 %)	4 (30.8%)	*0.38
3	53 (45.7%)	9 (69.2%)	
4	8 (6.9%)	0 (0%)	
pN			
0	26 (43.3%)	1 (16.7%)	
1	34 (56.7%)	5 (83.3%)	0.45
X	56	7	
pM			
1	21 (18.1%)	1 (7.7%)	0.70
X	95 (81.9%)	12 (92.3%)	
L			
0	42 (36.2%)	6 (46.2%)	0.55
1	74 (63.8%)	7 (53.8%)	
V			
0	52 (44.8%)	7 (53.8%)	0.57
1	64 (55.2%)	6 (46.2%)	
Pn			
0	59 (50.9%)	8 (61.5%)	0.56
1	57 (49.1%)	5 (38.5%)	
R			
0	44 (46.8%)	5 (50.0%)	
1	44 (46.8%)	5 (50.0%)	*1.00
2	6 (6.4%)	0 (0%)	
X	22	3	
Grading			
G1	5 (4.3%)	0 (0%)	
G2	73 (62.9%)	4 (30.8%)	***0.014**
G3	38 (32.8%)	9 (69.2%)	

Fisher's exact test, $\chi 2$ test or Mann-Whitney test (age) were used. *denotes NOS+mucinous+papillary+intestinal vs. adenosquamous+signet ring+solid; UICC 1 + 2 vs. 3 + 4; pT1+2 vs. 3 + 4; R0 vs. R1+2; G1 + 2 vs. G3. Abbreviations: GBC, gallbladder cancer; UICC, Union for International Cancer Control.
